# The Impact of the COVID-19 Pandemic on Elite Swimmers and Water Polo Players: Lessons for the Future

**DOI:** 10.3390/epidemiologia5020020

**Published:** 2024-06-18

**Authors:** Georgios Marinos, Dimitrios Lamprinos, Michail Papapanou, Anastasia Sofroni, Anastasia Papaioannou, Dionysios-Nikolaos Miletis, Paraskevi Deligiorgi, Kostas A. Papavassiliou, Gerasimos Siasos, Evangelos Oikonomou, George Rachiotis, Konstantinos Tsamakis, Dimitrios Schizas

**Affiliations:** 1 Department of Hygiene, Epidemiology and Medical Statistics, School of Medicine, National and Kapodistrian University of Athens, Agiou Thoma 17, 11527 Athens, Greece; 2 Emergency Care Department, Laiko General Hospital, 11527 Athens, Greece; dimitrislamprinos@gmail.com (D.L.); a.sofroni@hotmail.com (A.S.); evi_deligiorgi@hotmail.com (P.D.); 3 Second Department of Obstetrics and Gynecology, Medical School, “Aretaieion Hospital”, National and Kapodistrian University of Athens, 11528 Athens, Greece; mgpapapanou@gmail.com; 4 Health Center of Nea Makri, 19005 Attica, Greece; anpapai@yahoo.com; 5 Medical School, National and Kapodistrian University of Athens, 11527 Athens, Greece; dionisismiletis@gmail.com; 6 First Department of Respiratory Medicine, “Sotiria” Hospital, Medical School, National and Kapodistrian University of Athens, 11527 Athens, Greece; konpapav@med.uoa.gr; 7 First Department of Cardiology, Hippokration General Hospital, Medical School, National and Kapodistrian University of Athens, 11527 Athens, Greece; gsiasos@med.uoa.gr (G.S.); boikono@gmail.com (E.O.); 8 Third Department of Cardiology, Thoracic Diseases General Hospital Sotiria, Medical School, National and Kapodistrian University of Athens, 11527 Athens, Greece; 9 Department of Hygiene and Epidemiology, Faculty of Medicine, University of Thessaly, 41500 Larissa, Greece; grachiotis@gmail.com; 10 Second Department of Psychiatry, ‘Attikon’ University Hospital, National and Kapodistrian University of Athens, 12462 Athens, Greece; ktsamakis@gmail.com; 11 First Department of Surgery, National and Kapodistrian University of Athens, Laikon General Hospital, 11527 Athens, Greece; schizasad@gmail.com

**Keywords:** COVID-19, elite aquatic athletes, mental health, pandemic

## Abstract

Background: The COVID-19 pandemic has disrupted global daily life, including the world of elite athletes. This paper examines the multifaceted impact the COVID-19 pandemic had on elite swimmers and water polo athletes, specifically their mental health, their concerns over the virus, their intentions of getting vaccinated, and sleep disturbances that they may have faced. Methods: We conducted a cross-sectional study on elite swimmers and water polo players, using an anonymous questionnaire. Results: A total of 200 elite athletes participated. The majority of the participants reported a negative impact on their mental health, screened positive for insomnia (n = 107 (53.5%), with females (n = 101; 57.7%), swimmers (n = 100, 66.7%), and university students (n = 71, 71.7%) being more vulnerable (*p* < 0.001). Concerns about contracting the disease especially during important training or tournament periods and potential career disruption also affected their psychological well-being. While the majority (75%) had the intention of getting vaccinated, an alarming percentage was yet uncertain over its decision. Conclusions: This study highlights the significant psychological distress faced by elite aquatic athletes during the pandemic. It emphasizes the difficulties faced by elite swimmers and water polo athletes and determines not only the importance of addressing the vaccination intentions of athletes, but also how critical it is to confront the challenges they face both for their personal health and for the restoration of world sports to their pre-pandemic state. More large-scale studies are required to inform policies targeted at minimizing disruption to the athletes’ career, provision of information on preventive measures and vaccination, and improvement in psychological well-being in case of similar major public health issues in the future. Additionally, this study calls for further research to explore the unique challenges faced by aquatic athletes, such as those related to their training environments and fear of contagion, to better support them in future public health crises.

## 1. Introduction

The COVID-19 pandemic, a massive-scale global crisis, disrupted daily life, challenging societies, economies [1], and healthcare systems [2] worldwide. As humanity struggled with COVID-19, the world of sports, too, was facing a new threat [3]. In a domain where competition, training, and performance are part of everyday life, the pandemic introduced a deep disturbance, affecting not only athletes’ physical preparation but also their mental well-being, their careers [4,5], and even their attitudes toward vaccination [6,7].

Elite athletes represent a unique part within the broader spectrum of society. They dedicate their lives to the pursuit of physical excellence, undergoing rigorous training programs, enduring the demands of high-stakes competition [8]. This unique group of people is characterized by relentless dedication, intense pressure, and unwavering commitment, all of which converge to shape the lives and identities of these individuals. The COVID-19 pandemic disrupted this way of living in several ways, prompting a perhaps never-before-seen reevaluation of the intersection between athletic pursuits and global crises, which will need to be addressed in similar future events. While the world adjusted to lockdowns, social distancing, and remote work, elite athletes faced the challenge of maintaining their training regimes in a daily life characterized by uncertainty, event cancellations [3], and health-related anxieties [4,5]. The physical and mental obstacles of this lockdown period have been an ongoing subject of concern, both for the athletes themselves and the broader sports community [3], even long after its conclusion. Elite athletes are characterized for their mental resilience, but the pandemic forced even these individuals to confront psychological challenges. The sudden cessation of competitions, uncertainty about the resumption of sporting events, and concerns about contracting COVID-19 overshadowed the athletes’ lives. As a result, anxiety and worry affected them [4,5], with athletes struggling not only with the fear of infection but also with the potential destruction of their careers.

Sleep, a mandatory part of athletic recovery and performance, emerged as another crucial aspect of elite athletes’ lives affected by the pandemic. The disrupted training schedules, the uncertainty surrounding competitions, and the heightened stress levels created sleep disturbances. In the midst of unpredictability and uncertainty, the world turned to science for a solution: vaccines. Vaccination was the weapon in the fight against COVID-19, offering hope for a return to normalcy. Understanding vaccination intentions among elite athletes was not only a matter of individual choice but also held broader implications for public health and the resumption of sports events.

The recent literature highlights the multifaceted impact of the pandemic on athletes. Rassolnia and Nobari explored the influence of socio-economic status and physical activity on psychological well-being and sleep quality among college students, revealing significant disturbances during the pandemic period. Similarly, Mahdi Ashouri et al. examined the differential effects of COVID-19 on the lifestyles of vaccinated and unvaccinated elite athletes across various countries, emphasizing the varied responses and adaptations among athletes depending on their vaccination status. These studies underscore the complexity of the pandemic’s impact on athletes and highlight the need for targeted research to understand these implications better [9,10].

The aim of this cross-sectional study was to examine the impact of the COVID-19 pandemic on elite swimmers and water polo athletes, specifically their mental health, with a focus on insomnia, their concerns over the virus, and their intentions of getting vaccinated.

## 2. Materials and Methods

### 2.1. Study Design and Participants

We conducted a cross-sectional study on elite athletes visiting the Laikon General Hospital’s emergency department during the period of February to March 2021. We included elite swimmers and water polo athletes who voluntarily agreed to participate in the survey. Participants with severe and uncorrectable cognitive, visual, or hearing impairments that would hinder their ability to comprehend and respond to the survey were excluded. Each participant received an anonymous structured questionnaire (Appendix A Questionnaire). We stored all deidentified athlete responses in a Microsoft Excel file, which was accessed exclusively by the involved investigators. Verbal consent was obtained from each participant prior to their inclusion in the study. Participants provided anonymous informed consent by agreeing to voluntarily complete the questionnaire. The study adhered to the Declaration of Helsinki, and its protocol received approval from the N.S. Christeas Laboratory of Experimental Surgery and Surgical Research (protocol number: 2413-B/18-02-2021).

### 2.2. Questionnaire

#### 2.2.1. Demographics

The questionnaire included demographic items such as age group (i.e., ≤18, 19 to 23, 24 to 28, and >28), gender, level of education, sport, cohabitation status, and cohabitants.

#### 2.2.2. COVID-19 Status, Pandemic, and Career

Participants also answered general questions about their previous COVID-19 diagnosis status (i.e., yes, no, or unknown) and their anxiety regarding future infections (i.e., yes or no). Additionally, they indicated their COVID-19 vaccination status or intention to vaccinate (i.e., yes, no, or unknown) and reported their influenza vaccination status during the 2020–2021 period (i.e., yes or no). Participants also responded to questions related to the COVID-19 pandemic’s impact on their athletic career, progress, and goals. These questions, assessed on a 5-point Likert scale (ranging from “Not at all” to “Very much”), covered concerns about SARS-CoV-2 infection, training progress, changes in schedules or events, and career prospects during the pandemic.

#### 2.2.3. Psychological Well-Being and Insomnia

Athletes were asked whether they had sought expert mental health support during the pandemic (i.e., yes or no) and were questioned about their psychological well-being in the ten days preceding the questionnaire. They rated their feelings using a 5-point Likert scale, indicating how often they experienced feelings such as vividness, nervousness, depression, calmness, energy, sadness, exhaustion, happiness, and tiredness. The questionnaire also included a dedicated section on insomnia, using the Athens Insomnia Scale (AIS). Each AIS item could receive from 0 to 3 points, with insomnia defined as an AIS score ≥ 6, following the original recommendations by the tool’s developers [11].

#### 2.2.4. Data Validation

The questionnaire was pre-tested on a small group of elite athletes (n = 20) to identify any issues with clarity, relevance, and comprehension. Feedback from the pre-test was used to refine the questionnaire. The mental health assessment tools (SF-36 and AIS) used in the questionnaire have been validated in multiple populations and were selected for their established reliability and validity. Responses were checked for completeness and consistency. Any incomplete or inconsistent responses were reviewed and addressed where possible. Data were anonymized and stored securely to ensure confidentiality.

### 2.3. Statistical Analysis

All analyzed variables were categorical, and we expressed them as frequencies and percentages. To assess potential associations between insomnia (i.e., yes: AIS ≥ 6, no: AIS < 6) and intention for COVID-19 vaccination [i.e., positive, negative, or unknown, or known (either positive or negative) versus unknown], we used the Chi-square or Fisher’s exact tests [12]. We calculated crude odds ratios (ORs) with their corresponding 95% confidence intervals (95%CI) and *p*-values through univariate binary logistic regression, with insomnia and “intention for vaccination” as the dependent variables [13]. Regarding the “intention for vaccination” variable, we conducted sensitivity analyses, excluding participants with unknown vaccination intentions. We performed two separate analyses: one comparing the athletes with positive to those with negative or unknown intentions, and another comparing those with unknown and known intentions. All analyses were performed using the Stata Statistical Software, version 13 (StataCorp LP, College Station, TX, USA). We considered a two-tailed *p*-value < 0.05 as statistically significant.

## 3. Results

### 3.1. Participant Demographics

**Figure 1:**
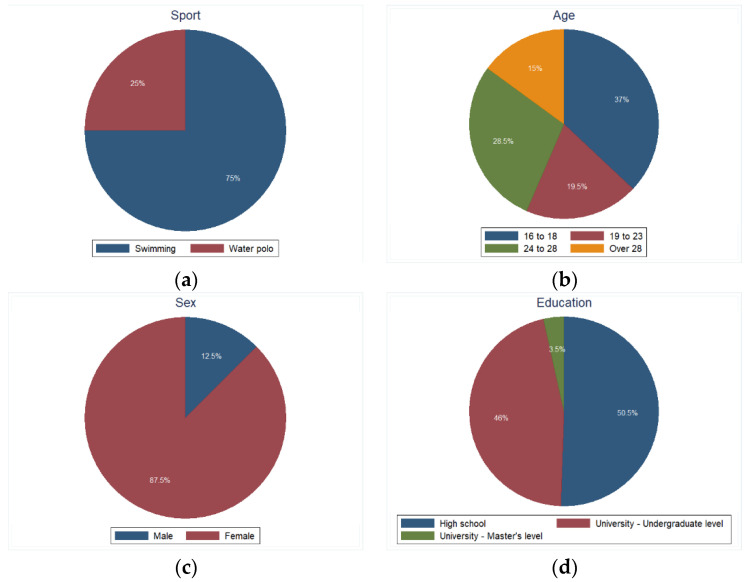

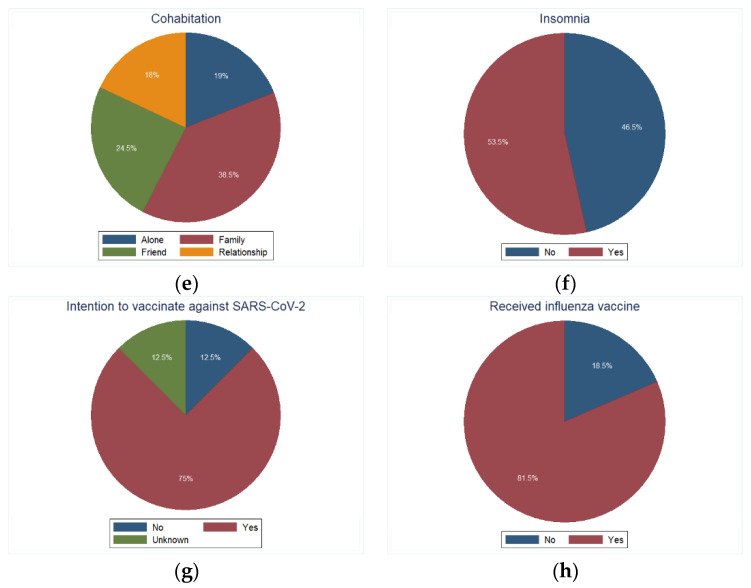
Pie charts of the main participants’ characteristics and outcomes. (**a**) Sport; (**b**) Age; (**c**) Sex; (**d**) Educational level; (**e**) Cohabitation; (**f**) Screened positive for insomnia as per the Athens Insomnia Scale; (**g**) Intended to vaccinate against SARS-CoV-2 (at least one dose); (**h**) Received the influenza vaccine during the 2020–2021 period.

A total of 200 elite athletes participated in this study, consisting of 150 (75.0%) swimmers and 50 (25.0%) water polo athletes. Most of the participants were in the 16–18 age group [74 (37.0%)], identified as female [175 (87.5%)], had a high-school educational level [101 (50.5%)], and were cohabiting [162 (81.0%)], with 87 (43.5%) living with one individual (Appendix B—Table A1, Figure 1).

### 3.2. COVID-19 Status, Pandemic, and Career

Only 28% of participants reported a prior positive SARS-CoV-2 status for themselves or their acquaintances. Furthermore, 47% expressed significant (i.e., much or very much) worry about contracting COVID-19, with even higher levels of concern during pre-Olympic training (71.5%), the Olympics (68.5%), or European/World tournaments (68.5%). About 59.5% were anxious about potential changes in their training schedules or events, 34% perceived substantial training progress loss during the pandemic, and many were greatly concerned about major career changes (67.5%) or the loss of career goals (65.5%) due to the pandemic (Appendix B—Table A2).

The majority of athletes (75.0%) expressed an intention to vaccinate against SARS-CoV-2, while 12.5% had unknown vaccination intentions at the time of the study. Notably, 81.5% had received the flu vaccine during the 2020–2021 period (Appendix B—Table A1, Figure 1). The intention for vaccination (positive, negative, or unknown) showed significant associations with sport (*p* = 0.005), age group, educational level, cohabitation status, the number and type of cohabitants, the need for expert mental health support, history of athlete’s or acquaintance’s SARS-CoV-2 positivity, and prior influenza vaccination (all *p* < 0.001, Appendix B—Table A3). In analyses restricted to participants with known (i.e., positive or negative) vaccination intentions, water polo athletes (OR 10.62, 95%CI 1.39 to 80.85), those in the 24–28 age group (OR 7.91, 95%CI 2.23 to 28.09), university-level athletes (OR 7.33, 95%CI 2.11 to 25.54), cohabiting athletes (OR 61.42, 95%CI 16.52 to 228.29), those with a history of COVID-19 positivity, and those with prior influenza vaccination (OR 111.71, 95%CI 23.80 to 524.28) were more likely to express a positive intention to vaccinate against COVID-19 (Appendix B—Table A4). The results of the sensitivity analyses comparing those with positive versus negative or unknown intentions, and those with unknown to athletes with known intentions, can be found in supplementary tables—Supplementary Table S1 and Supplementary Table S2, respectively.

### 3.3. Psychological Well-Being and Insomnia

The following rates of elite athletes reported feeling most or all of the time during the ten days preceding their participation in the study: vivid (44.5%), nervous (9.0%), so depressed that nothing could cheer them up (8.0%), peaceful (37.5%), energetic (44.5%), tired (54.0%), exhausted (33.0%), and sad (9.5%) (Appendix B—Table A5).

The majority of athletes [107 (53.5%)] screened positive for insomnia as per the AIS (Supplementary Table S3, Figure 1). The likelihood of experiencing insomnia, according to the AIS, was significantly higher in the 24 to 28 age group (OR 2.35, 95%CI 1.14 to 4.87) and the age group of athletes > 28 years (OR 14.00, 95%CI 3.11 to 63.07) compared to the 16 to 18 age group. Female athletes (OR 4.32, 95%CI 1.65 to 11.35), swimmers (OR 12.29, 95%CI 5.26 to 29.27), university-level athletes (OR 4.58, 95%CI 2.52 to 8.32), and those who themselves or whose close acquaintances had previously tested positive for COVID-19 (OR 15.40, 95%CI 6.14 to 38.63) were significantly more likely to screen positive for insomnia compared to their male counterparts, water polo athletes, individuals with a high-school level of education, and those without a history of COVID-19 positivity, respectively. Insomnia was also significantly associated with the number of cohabitants and the nature of the athletes’ relationships with these cohabitants (both *p* < 0.001). Finally, individuals with an unknown intention regarding COVID-19 vaccination were more likely to screen positive for insomnia than those with known (either positive or negative) intentions (OR 12.46, 95%CI 2.85 to 54.46, Table A6). Twenty percent of elite athletes sought expert mental health support during the pandemic (Appendix B—Table A1), and all of them tested positive for insomnia. However, 87 (81.3%) of those who screened positive for insomnia did not seek such mental health support during the pandemic (Appendix B—Table A6).

## 4. Discussion

This study records the experiences of elite swimmers and water polo athletes during the pandemic and the implications it had for both their lives and the world of sports. The psychological well-being of athletes affects not only their personal lives but also their performance and the integrity of sports as a whole. The evolution of the pandemic until its eventual dissolution showed how imperative recognizing and addressing the challenges faced by elite athletes is, in order to preserve their physical and mental health and ensure the safe resumption of sports events. Elite athletes invest countless hours in rigorous training and dedication to reach the peak of their sporting careers. The emergence of COVID-19 had brought a wave of uncertainties, increasing the already high-pressure environment they lived in. During the COVID-19 lockdown, the athletes’ training was taking place rarer and its duration was shorter, which could have been a factor contributing to higher depression, anxiety, and stress levels. A major cause is inspiration. Athletes are unsure about when the contests will reinstitute in the future and that could be the reason for the high percentage of athletes who mentioned productivity and mood disturbances. An extreme decrease in training load can affect their psychosocial engagement and could cause anatomical adaptations related to training (detraining) [14]. These alterations could be the reason for the increased risk of injury if training is not appropriate. Moreover, other factors of depressive symptoms are social isolation and the loneliness that are associated with decreased physical activity [15,16]. This study aims to uncover the psychological effect of the pandemic on these athletes. It analyzes their anxieties, worries, and concerns, which had been increased by the pandemic’s impact on training schedules, events, and, in some cases, the course of their entire athletic careers. Such information can be critical for the future preparation of sports events, especially under circumstances of public health concerns. Elite athletes bear with a lot of mental health symptoms and disorders in comparable or greater rates than those of non-athletes [17]. The mental stressors that affect athletes during the COVID-19 pandemic have been mentioned in latent publications [18,19,20]. The impact of the COVID-19 pandemic on dealing with mental health problems and disorders in athletes has received little attention.

There are management forethoughts for mental health symptoms and disorders in elite athletes. These options of management are various treatments such as outpatient psychotherapy and outpatient pharmacotherapy and other levels of care [21]. It is possible that these management strategies will obtain value into the foreseeable future because of the numerous waves of COVID-19.

There should be careful consideration of the pandemic’s influence on the way of managing those symptoms and disorders. The main lines of mental health management (psychotherapy, pharmacotherapy) remain. However, their use on athletes may be different during the pandemic. Moreover, management and diagnostic considerations for athletes can be applied to what we know about the pandemic, to make a theory of the impacts on athletes’ mental health and make appropriate recommendations [21]. The COVID-19 pandemic is an opportunity for researchers to find ways of managing mental health symptoms and disorders in elite athletes and maybe these methods can be used beyond the pandemic. For instance, elite athletes usually travel frequently, and they would take advantage of telehealth even after the end of the pandemic under certain circumstances. Athletes should keep having access to the resources necessary to participate in telehealth. There can be inconsistencies or improper consequences that might result from the use of telehealth and providers should be alert. Also, because of the fame of elite athletes around the world, the management of their mental telehealth should be confidential [21,22]. Moreover, insurance coverage would need to change for many athletes in order for them to participate in telehealth services in the future. Mental healthcare must be underlined, and future guidance should make providers of mental healthcare more capable of giving the best care to the athletes. Historically, there has been limited uptake of telehealth services, attributed in large part to providers’ unwillingness to adopt this modality [21].

Jaenes et al. found that home confinement led to significant changes in mood states and adaptive behaviors in Spanish swimmers, highlighting that aquatic athletes might face distinct psychological challenges compared to athletes from other sports. For example, the fear of contagion may be exacerbated by the close physical proximity required in water polo and the communal nature of swimming facilities. Additionally, the specific physical demands and training environments of aquatic sports, which are less easily replicated outside of professional facilities, may contribute to increased stress and anxiety levels among these athletes [23].

Integrating these findings, it becomes evident that the unique characteristics of aquatic sports, such as the necessity for specialized training environments and the close physical contact inherent in some aquatic disciplines, might have influenced the specific aspects of fear of contagion and other well-being issues observed during the pandemic. By contextualizing our results within the broader framework of the existing literature, we can better understand the nuanced impacts of the pandemic on aquatic athletes and develop more tailored support strategies for their mental and physical well-being during such crises.

A significant portion of participants reported feelings of sadness, exhaustion, and anxiety, highlighting the deep burden on their mental health. Alarmingly, a substantial number of athletes screened positive for insomnia, aligning with previous research indicating an increased prevalence of sleep disturbances among athletes in times of heightened stress. Particularly vulnerable were female athletes, swimmers, and those at university level.

At the beginning of the COVID-19 period, symptoms of anxiety and depression were increased in professional footballers, both males and females, mainly those who were anxious about their future as players [24]. Disturbed eating patterns (76%), depression (52%), and disturbed sleep patterns (79%) were mentioned by elite athletes from South Africa [25]. A web study at the beginning of the COVID-19 pandemic among Sweden’s top leagues in handball, soccer, and ice hockey showed that 70% of female athletes and 40% of male athletes mentioned feeling psychologically worse than before the pandemic. A total of 19% of female athletes mentioned increased symptoms of depression [26]. All things considered, there are indicators that underly the fact that the COVID-19 pandemic affected elite athletes’ mental health [27].

The results align with previous research that showed the important prevalence of insomnia during the pandemic. Preventive measures to find athletes suffering from insomnia and psychological distress are suggested, even more so if they do not seek assistance by themselves, as it can be detrimental to their training progress and career goals. The majority of participants expressed concerns about the risk of contracting COVID-19, with these concerns reaching their peak during critical training and tournament periods. These anxieties were further combined by the uncertainties surrounding changes in training schedules, competition events, and the potential disruption of their athletic careers.

In view of a study that examined the way of association between the COVID-19 pandemic situation and mental health problems, a notable risk factor for depression symptoms and anxiety was financial concerns. The above agree with the findings among football players, mainly those who are uncertain about their future [20]. Lower depression symptoms and insomnia appeared to those athletes who kept their daily routine [27].

According to a study, Norwegian athletes mentioned higher levels of depression than FIFAPRO athletes (females: 25.6% vs. 22% and males: 19.9% vs. 13%) [27]. Symptoms of depression and insomnia were the two main axes from the study and agree with the results from the research involving elite South African athletes [25,27]. Necessary factors for good mental health are both sleep quality and quantity. Consequently, insomnia symptoms could be an early sign of mental health problems [28]. Olympic and Paralympic athletes in the Norwegian population had a lower level of depression symptoms and anxiety than elite and semi-elite athletes. The reason why it happens could be that these groups of athletes have strong support available, and these athletes can have immediate access to psychological support [27]. These findings emphasize the vulnerability of specific athlete subgroups and the need for the future creation of tailored mental health support mechanisms. For those athletes who compete at international level, the loss of seasons that took place because of the pandemic was irreparable, because in many sports, the athletes’ fine form does not last for long [29,30]. Comparing these results with those from other populations and sports disciplines reveals both unique and common challenges faced by athletes during the pandemic. For instance, Rassolnia and Nobari found significant disturbances in psychological well-being and sleep quality among college students during the pandemic, influenced by socio-economic status and physical activity levels [9]. This aligns with our findings that elite aquatic athletes also experienced high levels of psychological distress and sleep disturbances, suggesting that these issues are common across different populations.

All things considered, the COVID-19 vaccine was a great chance for those athletes to come back into daily training and competing. In many countries around the world, elite athletes who are famous have a very important role in preventive health [31,32]. In Poland, the government called elite athletes to encourage people to get vaccinated, hoping that the power that elite athletes had could increase the vaccination rates. Olympic athletes called not only for the COVID-19 vaccine but also for equal access to vaccination for all people in the world [33]. Last but not least, myocarditis, pulmonary embolism, coagulopathy, and lung damage are some complications of COVID-19, which affect endurance athletes and they can prevent contracting them by getting vaccinated [34]. The incidence of myocarditis among the athletes’ population ranges from 1% to 4% according to a meta-analysis [35]. Athletes who had mild or asymptomatic COVID-19 infection had no signs of myocarditis but 19% of them revealed abnormalities by CMR [36]. It seems to be an association between vaccination and the vaccination of coaches, teammates, and family members. If most people in the environment are vaccinated, the athletes may feel pressure to get vaccinated [37]. Motivation for vaccination was associated with the acceptance of norms held by the reference team [6,38].

Mahdi Ashouri et al. examined the lifestyles of vaccinated and unvaccinated elite athletes and found that the pandemic had diverse impacts depending on vaccination status. Our study did not directly compare vaccinated and unvaccinated athletes, but the high willingness to get vaccinated (75%) among our participants indicates a proactive effort to reduce health risks [10]. Future research could delve deeper into how vaccination status influences mental health and training outcomes in elite athletes.

People’s attitudes toward vaccination are described in the sociological subdiscipline of social vaccinology and concern all the above mentioned [39]. Vaccination against COVID-19 is often a subject of social debate because it is perceived as a relatively harmless illness. If an athlete is influenced by people who adhere to this interpretation of the social world, the chance to get vaccinated decreases [36]. A significant association was found between the level of sport and anxiety that a severe infection of COVID-19 could exclude athletes from training (mainly among NAT athletes). The reason for the above maybe is that elite athletes are prone to infections for several reasons. First of all, they travel frequently and many times to countries with low rates of vaccination in the community. Also, sports tend to bring athletes into frequent contact with other athletes’ coaches and team members during training and competition time and the risk of infection by COVID-19 is high among elite athletes [40]. The reduction in the acquired immune response by intense exercise, known as “open window”, is the reason for the high risk of infections and mainly respiratory diseases [6].

Understanding the intention of vaccination against COVID-19 among elite athletes is crucial for the development of effective public health strategies. While a majority expressed an acceptance toward vaccination, a notable percentage had uncertain intentions. This study recorded the factors affecting vaccination decisions, revealing that sport type, age, education level, cohabitation status, COVID-19 history, and prior influenza vaccination all played a role.

### Limitations

It is essential to acknowledge the limitations of this study. The cross-sectional design, while providing valuable insights, restricts the establishment of causal relationships and the ability to capture the dynamic nature of athletes’ experiences during the pandemic. Furthermore, the reliance on self-report measures for psychological well-being and vaccination intention may introduce response bias and limit the precision of the data. Lastly, this study highlights the significant psychological impacts experienced by elite swimmers and water polo players during the COVID-19 pandemic. It is important to note that these findings are specific to these aquatic sports, and while they may suggest potential trends that could be observed in other sports, direct generalizations should be avoided. Future research should consider the adoption of longitudinal methodologies and the inclusion of more diverse athlete samples to provide a holistic understanding of the pandemic’s impact on elite athletes’ well-being.

## 5. Conclusions

The COVID-19 pandemic has left an indelible mark on the world of elite athletes, affecting their psychological well-being, sleep patterns, and vaccination intentions. This study, positioned where sports, psychology, and public health converge, emphasizes the unique challenges faced by elite swimmers and water polo athletes and underlines the urgent need for personalized support mechanisms. These findings are significant because they indicate that assisting athletes with alternative training regimes and continuing a regular sleeping pattern is essential as it might also affect the development of depression and vice versa. Moreover, the varying intentions regarding COVID-19 vaccination among athletes highlight that specific public health strategies should be developed to address the vaccination intentions of athletes, taking into account the factors that influence their decisions. While the findings provide valuable insights, it is crucial to state that they are specific to these groups of elite athletes. Only with further research can it be determined if athletes in other sports experience similar impacts. Therefore, this article suggests a cautious approach when extending these findings beyond the studied sample. More high-quality, large-scale studies are necessary to inform policies targeted at minimizing the disruption to the athletes’ career, provision of information on preventive measures and vaccination, and improvement in their psychological well-being in case of similar major public health issues in the future.

## Data Availability

The study data are available from the corresponding author upon reasonable request.

## Supplementary Materials

The following supporting information can be downloaded at: https://www.mdpi.com/article/10.3390/epidemiologia5020020/s1, Table S1: comparison between respondents intending, and those with no or unknown intention to receive vaccination against the SARS-CoV-2. The fourth column contains the *p*-values of the Chi-square tests while the fifth column indicates the crude odds ratios and 95% confidence intervals as these derive from the univariate binary logistic regression; Table S2: comparison between respondents with known and those with unknown intention to receive vaccination against the SARS-CoV-2. This is a sensitivity analysis investigating how exclusion of those with unknown intention affects the results. The fourth column contains the *p*-values of the Chi-square tests while the fifth column indicates the odds ratios and 95% confidence intervals as these result from the univariate binary logistic regression; Table S3: responses of the elite athletes to questions from the Athens Insomnia Scale.

## Appendix A

            **General Hospital Laiko**

              **Questionnaire**

          **PLEASE READ CAREFULLY.**

Please answer all questions without leaving any out.

Only one answer is possible for each question.

YOUR ANSWERS WILL BE KEPT STRICTLY CONFIDENTIAL.

Your participation is voluntary and anonymous. At any point in the investigation, you will

you can withdraw your participation.

**Please write**

Type of sport ……………………………………………………………

1. Age 16–18   24–28
       19–23   28>
2. Gender Male   Female
3. Education   Primary school   Private university   MSc
           Secondary education  Technical School    PhD
           High school     University
4. **During the restrictive measures, with whom do you live in the same house?**

Alone
With my family
Friend
My partner
Other:

5. **How many people live with you in the same house?**

Alone
Me & 1 other person
Me & 2 other people
Me & 3 or more people

6. **Have you, a family member or a friend been diagnosed with COVID-19?**

Yes
No
I do not know

7. **Are you worried that you, a family member, or a friend might get COVID-19?**

Not at all
A little bit
Moderate
Very
Very much

The following questions refer to how you feel and how things have been going with you in the last 10 days. For each question, please give the answer that comes closest to how you felt.

8. **Did you feel full of life?**

Not at all
A few times
Sometimes
Most of the time
Always

9. **Did you have a lot of irritation?**

Not at all
A few times
Sometimes
Most of the time
Always

10. **Have you ever felt so down, that nothing could cheer you up?**

Not at all
A few times
Sometimes
Most of the time
Always

11. **Did you feel calm and peaceful?**

Not at all
A few times
Sometimes
Most of the time
Always

12. **Did you have a lot of energy?**

Not at all
A few times
Sometimes
Most of the time
Always

13. **Have you been feeling moody and gloomy?**

Not at all
A few times
Sometimes
Most of the time
Always

14. **Were you feeling exhausted?**

Not at all
A few times
Sometimes
Most of the time
Always

15. **Were you feeling happy?**

Not at all
A few times
Sometimes
Most of the time
Always

16. **Were you feeling tired?**

Not at all
A few times
Sometimes
Most of the time
Always

This scale is intended to record your own assessment of the difficulties you may have experienced in sleeping. Please select (by circling the appropriate number) the questions that indicate in your estimation the degree of difficulty, provided that they occurred at least three times per week during the past month.

17. **Sleep induction (time you need to fall asleep after lights out)**

0 No problem
1 Slight delay
2 Significant delay
3 Too late or you didn’t sleep at all

18. **Awakenings during the night**

0 No problem
1 Slight delay
2 Significant delay
3 Too late or you didn’t sleep at all

19. **Final awakening earlier than desired**

0 Not earlier
1 A little earlier
2 Significantly earlier
3 You didn’t sleep much earlier or at all

20. **Total sleep duration**

0 Adequate
1 Slightly inadequate
2 Significantly inadequate
3 Very little or no sleep at all

21. **Overall quality of sleep**

0 Satisfactory
1 Slightly Unsatisfactory
2 Significantly Unsatisfactory
3 very unsatisfactory or you did not sleep at all

22. **Sense of well****-being during the day**

0 Normal
1 Slightly reduced
2 reduced
3 Significantly reduced

23. **Functioning during the day**

0 Normal
1 Slightly reduced
2 reduced
3 Significantly reduced

24. **Sleepiness during the day**

0 Not at all
1 Mild
2 Enough
3 Intense

25. **Are you worried about losing your athletic form?**

Not at all
A little bit
Moderate
Very much
Absolutely

26. **Are you worried about changes to your sports training and competition schedule?**

Not at all
A little bit
Moderate
Very much
Absolutely

27. **Are you worried about getting sick with COVID-19 during the preparation for the pre-Olympic tournaments?**

Not at all
A little bit
Moderate
Very much
Absolutely

28. **Are you worried about getting sick with COVID-19 during the preparation for the European or World Championships?**

Not at all
A little bit
Moderate
Very much
Absolutely

29. **Are you worried about a family member or friend getting sick with COVID-19 during the preparations for the pre-Olympic tournaments?**

Not at all
A little bit
Moderate
Very much
Absolutely

30. **Are you worried about contracting COVID-19 during the Olympics?**

Not at all
A little bit
Moderate
Very much
Absolutely

31. **Are you worried about contracting COVID-19 during the European or World Championships?**

Not at all
A little bit
Moderate
Very much
Absolutely

32. **Are you worried about missing out on athletic goals due to the COVID-19 pandemic?**

Not at all
A little bit
Moderate
Very much
Absolutely

33. **Are you worried about negative changes in your sports career due to the COVID-19 pandemic?**

Not at all
A little bit
Moderate
Very much
Absolutely

34. **Are you worried about loss of income due to the COVID-19 pandemic?**

A little bit
Moderate
Very much
Absolutely

35. **During the Pandemic, did you ask for the help of a healthcare professional?**
  Yes  No

36. **Are you willing to do be vaccinated against COVID-19?**
  Yes  No    I don’t know

37. **Have you been vaccinated against influenza virus for the season 2020-2021?**
  Yes  No

## Appendix B

**Table A1:** Characteristics of the respondents.

Variable	Responses
**Sport**	Swimming: 150 (75.0%) Water polo: 50 (25.0%)
**Age**	16 to 18: 74 (37.0%) 19 to 23: 39 (19.5%) 24 to 28: 57 (28.5%) >28: 30 (15.0%)
**Sex**	Male: 25 (12.5%) Female: 175 (87.5%)
**Educational level**	High school: 101 (50.5%) University—Undergraduate level: 92 (46.0%) University—Master’s level: 7 (3.5%)
**Cohabitation**	No: 38 (19.0%) Yes: 162 (81.0%)
**Number of cohabitants**	None: 38 (19.0%) One: 87 (43.5%) Two: 39 (19.5%) Three: 36 (18.0%)
**Cohabitants**	None: 38 (19.0%) Family members: 77 (38.5%) Friends: 49 (24.5%) Relationship: 36 (18.0%)
**Sought expert mental health support during the pandemic**	No: 180 (90.0%) Yes: 20 (10.0%)
**Athlete or close acquaintance priorly positive for SARS-CoV-2**	No: 131 (65.5%) Yes: 56 (28.0%) Unknown: 13 (6.5%)
**Intention for COVID-19 vaccination (at least one dose)**	No: 25 (12.5%) Yes: 150 (75.0%) Unknown: 25 (12.5%)
**Received the flu vaccine during the 2020–2021 period**	No: 37 (18.5%) Yes: 163 (81.5%)

**Table A2:** Responses of the elite athletes to questions on their self-reported anxiety about the COVID-19 pandemic itself as well as their self-reported anxiety about potential loss of career progress or major events due to the pandemic. The athletes responded on a 5-point Likert scale (0 = Not at all, 1 = A little, 2 = Somewhat, 3 = Much, 4 = Very much).

Question/Item	5-Point Likert Scale [N (%)]				
	Not at All	A Little	Somewhat	Much	Very Much
**Worried about becoming positive for COVID-19**	6 (3.0%)	31 (15.5%)	69 (34.5%)	69 (34.5%)	25 (12.5%)
**Perception of loss of training progress during the pandemic**	25 (12.5%)	63 (31.5%)	44 (22.0%)	68 (34.0%)	0 (0%)
**Worried about changes in training schedule or events**	6 (3.0%)	25 (12.5%)	50 (25.0%)	94 (47.0%)	25 (12.5%)
**Worried about COVID-19 positivity during training for the pre-Olympics**	13 (6.5%)	19 (9.5%)	25 (12.5%)	69 (34.5%)	74 (37.0%)
**Worried about COVID-19 positivity during the Olympics**	18 (9.0%)	19 (9.5%)	26 (13.0%)	48 (24.0%)	89 (44.5%)
**Worried about COVID-19 positivity** **during European or World tournaments**	13 (6.5%)	19 (9.5%)	31 (15.5%)	56 (28.0%)	81 (40.5%)
**Worried about loss of career** **goals due to COVID-19 pandemic**	6 (3.0%)	19 (9.5%)	44 (22.0%)	75 (37.5%)	56 (28.0%)
**Worried about major career changes due** **to COVID-19 pandemic**	3 (1.5%)	31 (15.5%)	31 (15.5%)	77 (38.5%)	58 (29.0%)

**Table A3:** Comparison between respondents intending, those not intending, and those with unknown intention to receive vaccination against the SARS-CoV-2. The fifth column contains the *p*-values of the Chi-square/Fisher’s exact tests.

Variable		Intention for Vaccination [n = 150 (75%)]	No Intention for Vaccination [n = 25 (12.5%)]	Unknown Intention for Vaccination [n = 25 (12.5%)]	*p*-Value
**Sport**	Swimming	104 (69.3%)	24 (16.0%)	22 (14.7%)	0.005
	Water polo	46 (92.0%)	1 (2.0%)	3 (6.0%)	
**Age**	16 to 18	50 (67.6%)	22 (29.7%)	2 (2.7%)	<0.001
	19 to 23	39 (100.0%)	0 (0%)	0 (0%)	
	24 to 28	54 (94.7%)	3 (5.3%)	0 (0%)	
	>28	7 (23.3%)	0 (0%)	23 (76.7%)	
**Sex**	Male	22 (88.0%)	1 (4.0%)	2 (8.0%)	0.323
	Female	128 (73.1%)	24 (13.7%)	23 (13.2%)	
**Educational level**	High School	75 (74.2%)	22 (21.8%)	4 (4.0%)	<0.001
	University	75 (75.8%)	3 (3.0%)	21 (21.2%)	
**Cohabitation**	No	16 (42.1%)	22 (57.9%)	0 (0%)	<0.001
	Yes	134 (82.7%)	3 (1.9%)	25 (15.4%)	
**Number of cohabitants**	None	16 (42.1%)	22 (57.9%)	0 (0%)	<0.001
	One	62 (71.3%)	1 (1.1%)	24 (27.6%)	
	Two	39 (100.0%)	0 (0%)	0 (0%)	
	Three	33 (91.7%)	2 (5.5%)	1 (2.8%)	
**Cohabitants**	None	16 (42.1%)	22 (57.9%)	0 (0%)	<0.001
	Family members	73 (94.8%)	2 (2.6%)	2 (2.6%)	
	Friends	46 (93.9%)	1 (2.0%)	2 (4.1%)	
	Relationship	15 (41.7%)	0 (0%)	21 (58.3%)	
**Sought expert mental health support during the pandemic**	No	150 (83.3%)	25 (13.9%)	5 (2.8%)	<0.001
	Yes	0 (0%)	0 (0%)	20 (100.0%)	
**Athlete or close acquaintance priorly positive for SARS-CoV-2**	No	104 (79.4%)	25 (19.1%)	2 (1.5%)	<0.001
	Yes	44 (78.6%)	0 (0%)	12 (21.4%)	
**Received the flu vaccine (2020–2021)**	No	14 (37.8%)	23 (62.2%)	0 (0%)	<0.001
	Yes	136 (83.5%)	2 (1.2%)	25 (15.3%)	

**Table A4:** Comparison between respondents intending and those not intending to receive vaccination against the SARS-CoV-2 after exclusion of respondents with unknown intention. This is a sensitivity analysis investigating how exclusion of those with unknown intention affects the results. The fourth column contains the *p*-values of the Chi-square tests while the fifth column indicates the odds ratios and 95% confidence intervals as these result from the univariate binary logistic regression.

Variable		Intention for Vaccination [n = 150 (85.7%)]	No Intention for Vaccination [n = 25 (14.3%)]	*p*-Value	OR (95% CI)	*p*-Value (OR)
**Sport**	Swimming	104 (81.4%)	24 (8.6%)	0.005	Ref.	-
	Water polo	46 (97.9%)	1 (2.1%)		10.62 (1.39 to 80.85)	0.023
**Age**	16 to 18	50 (69.4%)	22 (30.6%)	<0.001	Ref.	-
	19 to 23	39 (100.0%)	0 (0%)		-	-
	24 to 28	54 (94.7%)	3 (5.3%)		7.91 (2.23 to 28.09)	0.001
	>28	7 (100.0%)	0 (0%)		-	-
**Sex**	Male	22 (95.7%)	1 (4.3%)	0.206	Ref.	-
	Female	128 (84.2%)	24 (15.8%)		0.24 (0.03 to 1.88)	0.176
**Educational level**	High School	75 (77.3%)	22 (22.7%)	<0.001	Ref.	-
	University	75 (96.2%)	3 (3.8%)		7.33 (2.11 to 25.54)	0.002
**Cohabitation**	No	16 (42.1%)	22 (57.9%)	<0.001	Ref.	
	Yes	134 (97.8%)	3 (2.2%)		61.42 (16.52 to 228.29)	<0.001
**Number of cohabitants**	None	16 (42.1%)	22 (57.9%)	<0.001	Ref.	-
	One	62 (98.4%)	1 (1.6%)		85.25 (10.67 to 681.02)	<0.001
	Two	39 (100.0%)	0 (0%)		-	-
	Three	33 (94.3%)	2 (5.7%)		22.69 (4.74 to 108.60)	<0.001
**Cohabitants**	None	16 (42.1%)	22 (57.9%)	<0.001	Ref.	-
	Family members	73 (97.3%)	2 (2.7%)		50.19 (10.70 to 235.36)	<0.001
	Friends	46 (97.9%)	1 (2.1%)		63.25 (7.88 to 507.90)	<0.001
	Relationship	15 (100.0%)	0 (0%)		-	-
**Athlete or close acquaintance priorly positive for SARS-CoV-2**	No	104 (80.6%)	25 (19.4%)	0.002	Ref.	-
	Yes	44 (100.0%)	0 (0%)		-	-
**Received the flu vaccine (2020–2021)**	No	14 (37.8%)	23 (62.2%)	<0.001	Ref.	-
	Yes	136 (98.6%)	2 (1.4%)		111.71 (23.80 to 524.28)	<0.001

**Table A5:** Responses of the elite athletes to questions on their self-reported psychological well-being during the last ten days before answering the questionnaire. The athletes responded on a 5-point Likert scale (0 = None of the time, 1 = A little of the time, 2 = Some of the time, 3 = Most of the time, 4 = All of the time).

Question/Item	5-Point Likert Scale [N (%)]				
	None of the Time	A Little of the Time	Some of the Time	Most of the Time	All of the Time
**Feel vivid**	0 (0%)	31 (15.5%)	80 (40.0%)	75 (37.5%)	14 (7.0%)
**Feel nervous**	38 (19.0%)	75 (37.5%)	69 (34.5%)	18 (9.0%)	0 (0%)
**Feel so depressed that nothing can cheer them up**	70 (35.0%)	76 (38.0%)	38 (19.0%)	16 (8.0%)	0 (0%)
**Feel calm and peaceful**	20 (10.0%)	35 (17.5%)	70 (35.0%)	55 (27.5%)	20 (10.0%)
**Feel energetic**	7 (3.5%)	28 (14.0%)	76 (38.0%)	86 (43.0%)	3 (1.5%)
**Feel sad**	44 (22.0%)	72 (36.0%)	65 (32.5%)	14 (7.0%)	5 (2.5%)
**Feel exhausted**	15 (7.5%)	50 (25.0%)	69 (34.5%)	66 (33.0%)	0 (0%)
**Feel happy**	6 (3.0%)	13 (6.5%)	61 (30.5%)	99 (49.5%)	21 (10.5%)
**Feel tired**	4 (2.0%)	21 (10.5%)	67 (33.5%)	101 (50.5%)	7 (3.5%)

**Table A6:** Comparison between respondents with and those without insomnia as per the Athens Insomnia Scale (and a cut-off of 6). The fourth column contains the *p*-values of the Chi-square tests while the fifth column indicates the crude odds ratios and 95% confidence intervals as these result from the univariate binary logistic regression.

Variable		Insomnia [AIS ≥ 6, N = 107 (53.5%)]	No Insomnia [AIS < 6, N = 93 (46.5%)]	*p*-Value	OR (95% CI)	*p*-Value (OR)
**Sport**	Swimming	100 (66.7%)	50 (33.3%)	<0.001	Ref.	-
	Water polo	7 (14.0%)	43 (86.0%)		0.08 (0.03 to 0.19)	<0.001
**Age**	16 to 18	37 (50.0%)	37 (50.0%)	<0.001	Ref.	-
	19 to 23	2 (5.1%)	37 (94.9%)		0.05 (0.01 to 0.24)	<0.001
	24 to 28	40 (70.2%)	17 (29.8%)		2.35 (1.14 to 4.87)	0.021
	>28	28 (93.3%)	2 (6.7%)		14.00 (3.11 to 63.07)	0.001
**Sex**	Male	6 (24.0%)	19 (76.0%)	0.002	Ref.	-
	Female	101 (57.7%)	74 (42.3%)		4.32 (1.65 to 11.35)	0.003
**Educational level**	High School	36 (35.6%)	65 (64.4%)	<0.001	Ref.	-
	University	71 (71.7%)	28 (28.3%)		4.58 (2.52 to 8.32)	<0.001
**Cohabitation**	No	21 (55.3%)	17 (44.7%)	0.809	Ref.	
	Yes	86 (53.1%)	76 (46.9%)		0.92 (0.45 to 1.86)	0.809
**Number of cohabitants**	None	21 (55.3%)	17 (44.7%)	<0.001	Ref.	-
	One	47 (54.0%)	40 (46.0%)		0.95 (0.44 to 2.05)	0.898
	Two	9 (23.1%)	30 (76.9%)		0.24 (0.09 to 0.65)	0.005
	Three	30 (83.3%)	6 (16.7%)		4.05 (1.37 to 11.98)	0.012
**Cohabitants**	None	21 (55.3%)	17 (44.7%)	<0.001	Ref.	-
	Family members	18 (23.4%)	59 (76.6%)		0.25 (0.11 to 0.57)	0.001
	Friends	36 (73.5%)	13 (26.5%)		2.24 (0.91 to 5.52)	0.079
	Relationship	32 (88.9%)	4 (11.1%)		6.48 (1.91 to 21.94)	0.003
**Sought expert mental health support during the pandemic**	No	87 (48.3%)	93 (51.7%)	<0.001	Ref.	-
	Yes	20 (100.0%)	0 (0%)		-	-
**Athlete or close acquaintance priorly positive for SARS-CoV-2**	No	46 (35.1%)	85 (64.9%)	<0.001	Ref.	-
	Yes	50 (89.3%)	6 (10.7%)		15.40 (6.14 to 38.63)	<0.001
**Intention for COVID-19 vaccination (at least one dose)**	No	12 (48.0%)	13 (52.0%)	<0.001	Ref.	-
	Yes	72 (48.0%)	78 (52.0%)		1.00 (0.43 to 2.33)	1.000
	Unknown	23 (92.0%)	2 (8.0%)		12.46 (2.41 to 64.49)	0.003
**Intention for COVID-19 vaccination (at least one dose)**	Known	84 (48.0%)	91 (52.0%)	<0.001	Ref.	-
	Unknown	23 (92.0%)	2 (8.0%)		12.46 (2.85 to 54.46)	0.001
**Received the flu vaccine (2020–2021)**	No	21 (56.8%)	16 (43.2%)	0.660	Ref.	-
	Yes	86 (52.8%)	77 (47.2%)		0.85 (0.41 to 1.75)	0.660
