# Supplementary material for: The Impact of the COVID-19 Pandemic on Elite Swimmers and Water Polo Players: Lessons for the Future
Source: Epidemiologia (Basel). 2024 Jun 18;5(2):289–308. doi: 10.3390/epidemiologia5020020 (PMC11203378; doi:10.3390/epidemiologia5020020)
Supplement: Supplementary file 1 [file epidemiologia-05-00020-s001.zip › epidemiologia-3030808-supplementary.pdf]

Table S1. Comparison between respondents intending, and those with no or unknown intention to receive vaccination against the SARS-CoV-2. The fourth column contains the p-values of the Chi-square tests while the fifth column indicates the crude odds ratios and 95% confidence intervals as these derive from the univariate binary logistic regression.

Variable		Intention for vaccination	No or unknown intention for vaccination	*p*-value	OR (95% CI)	*p*-value (OR)
**Sport**	Swimming	104 (69.3%)	46 (30.7%)	**0.001**	Ref.	-
	Water polo	46 (92.0%)	4 (8.0%)		**5.09 (1.73 to 14.96)**	0.003
**Age**	16 to 18	50 (67.6%)	24 (32.4%)	**< 0.001**	Ref.	-
	19 to 23	39 (100.0%)	0 (0%)		-	-
	24 to 28	54 (94.7%)	3 (5.3%)		**8.64 (2.45 to 30.47)**	0.001
	> 28	7 (23.3%)	23 (76.7%)		**0.15 (0.06 to 0.39)**	< 0.001
**Sex**	Male	22 (88.0%)	3 (12.0%)	0.109	Ref.	-
	Female	128 (73.1%)	47 (26.9%)		0.37 (0.11 to 1.30)	0.121
**Educational level**	High School	75 (74.2%)	26 (25.8%)	0.806	Ref.	-
	University	75 (75.8%)	24 (24.2%)		1.08 (0.57 to 2.06)	0.807
**Cohabitation**	No	16 (42.1%)	22 (57.9%)	**<0.001**	Ref.	
	Yes	134 (82.7%)	28 (17.3%)		**6.58 (3.07 to 14.10)**	< 0.001
**Number of cohabitants**	None	16 (42.1%)	22 (57.9%)	**< 0.001**	Ref.	-
	One	62 (71.3%)	25 (28.7%)		**3.41 (1.54 to 7.54)**	0.002
	Two	39 (100.0%)	0 (0%)		**-**	-
	Three	33 (91.7%)	3 (8.3%)		**15.13 (3.94 to 58.11)**	< 0.001
**Cohabitants**	None	16 (42.1%)	22 (57.9%)	**< 0.001**	Ref.	-
	Family members	73 (94.8%)	4 (5.2%)		**25.09 (7.60 to 82.89)**	< 0.001
	Friends	46 (93.9%)	3 (6.1%)		**21.08 (5.56 to 80.01)**	< 0.001
	Relationship	15 (41.7%)	21 (58.3%)		0.98 (0.39 to 2.47)	0.970
**Athlete or close acquaintance priorly positive for**	No	104 (79.4%)	27 (20.6%)	0.900	Ref.	-
	Yes	44 (78.6%)	12 (21.4%)		0.95 (0.44 to 2.05)	0.900

**SARS-CoV-2**						
**Received the flu vaccine (2020-2021)**	No	14 (37.8%)	23 (62.2%)	**< 0.001**	Ref.	-
	Yes	136 (83.5%)	27 (16.5%)		**8.28 (3.78 to 18.09)**	< 0.001

Table S2. Comparison between respondents with known and those with unknown intention to receive vaccination against the SARS-CoV-2. This is a sensitivity analysis investigating how exclusion of those with unknown intention affects the results. The fourth column contains the p-values of the Chi-square tests while the fifth column indicates the odds ratios and 95% confidence intervals as these result from the univariate binary logistic regression.

**Variable**		**Unknown intention for vaccination**	**Known intention for vaccination**	***p*-value**	**OR (95% CI)**	***p*-value (OR)**
**Sport**	Swimming	22 (14.7%)	128 (85.3%)	0.109	Ref.	-
	Water polo	3 (6.0%)	47 (94.0%)		0.37 (0.11 to 1.30)	0.121
**Age**	16 to 18	2 (2.7%)	72 (97.3%)	**< 0.001**	Ref.	-
	19 to 23	0 (0%)	39 (100.0%)		-	-
	24 to 28	0 (0%)	57 (100.0%)		-	-
	> 28	23 (76.7%)	7 (23.3%)		**118.29 (22.94 to 609.84)**	< 0.001
**Sex**	Male	2 (8.0%)	23 (92.0%)	0.746	Ref.	-
	Female	23 (13.2%)	152 (86.8%)		1.74 (0.38 to 7.88)	0.472
**Educational level**	High School	4 (4.0%)	97 (96.0%)	**< 0.001**	Ref.	-
	University	21 (21.2%)	78 (78.8%)		**6.53 (2.15 to 19.81)**	0.001
**Cohabitation**	No	0 (0%)	38 (100.0%)	**0.005**	Ref.	-
	Yes	25 (15.4%)	137 (84.6%)		-	-
**Number of cohabitants**	None	0 (0%)	38 (100.0%)	**< 0.001**	-	-
	One	24 (27.6%)	63 (72.4%)		Ref.	-
	Two	0 (0%)	39 (100.0%)		-	-
	Three	1 (2.8%)	35 (97.2%)		**0.08 (0.01 to 0.58)**	0.013
**Cohabitants**	None	0 (0%)	38 (100.0%)	**< 0.001**	-	-
	Family members	2 (2.6%)	75 (97.4%)		Ref.	-
	Friends	2 (4.1%)	47 (95.9%)		1.60 (0.22 to 11.72)	0.646

	Relationship	21 (58.3%)	15 (41.7%)		**52.50 (11.11 to 248.03)**	< 0.001
**Athlete or close acquaintance priorly positive for SARS-CoV-2**	No	2 (1.5%)	129 (98.5%)	**< 0.001**	Ref.	-
	Yes	12 (21.4%)	44 (78.6%)		**17.59 (3.79 to 81.69)**	< 0.001
**Received the flu vaccine (2020-2021)**	No	0 (0%)	37 (100.0%)	**0.006**	Ref.	-
	Yes	25 (15.3%)	138 (84.7%)		-	-

Table S3. Responses of the elite athletes to questions from the Athens Insomnia Scale.

Question/Item	Responses [N (%)]
**Sleep induction**	Normal: 39 (19.5%)<br>Mild issue: 115 (57.5%)<br>Moderate issue: 40 (20.0%)<br>Strong issue: 6 (3.0%)
**Awaking during night**	No problem: 125 (62.5%)<br>Minor problem: 75 (37.5%)<br>Considerable problem: 0 (0%)<br>Serious problem or did not sleep at all: 0 (0%)
**Final awakening earlier than desired**	Not earlier: 120 (60.0%)<br>A little earlier: 80 (40.0%)<br>Markedly earlier: 0 (0%)<br>Much earlier or did not sleep at all: 0 (0%)
**Total sleep duration**	Sufficient: 36 (18.0%)<br>Slightly insufficient: 120 (60.0%)<br>Markedly insufficient: 38 (19.0%)<br>Very insufficient or did not sleep at all: 6 (3.0%)

**Overall quality of sleep (no matter how long they slept)**	Satisfactory: 82 (41.0%)<br>Slightly unsatisfactory: 110 (55.0%)<br>Markedly unsatisfactory: 8 (4.0%)<br>Very unsatisfactory: 0 (0%)
**Sense of well-being during the day**	Normal: 69 (34.5%)<br>Slightly decreased: 89 (44.5%)<br>Markedly decreased: 36 (18.0%)<br>Very decreased: 6 (3.0%)
**Functioning (physical and mental) during the day**	Normal: 117 (58.5%)<br>Slightly decreased: 44 (22.0%)<br>Markedly decreased: 34 (17.0%)<br>Very decreased: 5 (2.5%)
**Sleepiness during the day**	None: 58 (29.0%)<br>Mild: 122 (61.0%)<br>Considerable: 20 (10.0%)<br>Intense: 0 (0%)
**Insomnia (AIS Score ≥ 6)**	No: 93 (46.5%)<br>Yes: 107 (53.5%)
